# Dysregulated lipolysis and lipophagy in lipid droplets of macrophages from high fat diet‐fed obese mice

**DOI:** 10.1111/jcmm.17513

**Published:** 2022-08-13

**Authors:** Yohannes Getiye, Tatiana Angel Rice, Brandon D. Phillips, Daniel Fidel Carrillo, Guanglong He

**Affiliations:** ^1^ School of Pharmacy, College of Health Sciences University of Wyoming Laramie Wyoming USA

**Keywords:** autophagy, CARD9, DAG‐PKCδ, lipid droplet, lipolysis, lipophagy

## Abstract

Obesity is associated with lipid droplet (LD) accumulation, dysregulated lipolysis and chronic inflammation. Previously, the caspase recruitment domain‐containing protein 9 (CARD9) has been identified as a potential contributor to obesity‐associated abnormalities including cardiac dysfunction. In the current study, we explored a positive feedback signalling cycle of dysregulated lipolysis, CARD9‐associated inflammation, impaired lipophagy and excessive LD accumulation in sustaining the chronic inflammation associated with obesity. C57BL/6 WT and CARD9^−/−^ mice were fed with normal diet (ND, 12% fat) or a high fat diet (HFD, 45% fat) for 5 months. Staining of LDs from peritoneal macrophages (PMs) revealed a significant increase in the number of cells with LD and the number of LD per cell in the HFD‐fed WT but not CARD9^−/−^ obese mice. Rather, CARD9 KO significantly increased the mean LD size. WT obese mice showed down regulation of lipolytic proteins with increased diacylglycerol (DAG) content, and CARD9 KO normalized DAG with restored lipolytic protein expression. The build‐up of DAG in the WT obese mice is further associated with activation of PKCδ, NF‐κB and p38 MAPK inflammatory signalling in a CARDD9‐dependent manner. Inhibition of adipose triglyceride lipase (ATGL) by Atglistatin (Atg) resulted in similar effects as in CARD9^−/−^ mice. Interestingly, CARD9 KO and Atg treatment enhanced lipophagy. In conclusion, HFD feeding likely initiated a positive feedback signalling loop from dysregulated lipolysis, CARD9‐dependent inflammation, impaired lipophagy, to excessive LD accumulation and sustained inflammation. CARD9 KO and Atg treatment protected against the chronic inflammation by interrupting this feedforward cycle.

## 1 INTRODUCTION

Lipid droplets (LDs) are dynamic cytoplasmic organelles present in almost all cell types as a storage of neutral lipids.^1^ Besides its versatile roles in many biological processes, LD hypertrophy and hyperplasia are known key pathological features of obesity.^2^, ^3^, ^4^ Obesity is characterised by chronic low‐grade inflammation that leads to pro‐inflammatory conditions and metabolic diseases, such as insulin resistance, type II diabetes, atherosclerosis and cardiovascular disorders.^5^, ^6^, ^7^ In the progression of this chronic inflammatory condition, macrophages are the predominant cell type infiltrated into target tissues to release pro‐inflammatory mediators.^5^, ^8^, ^9^ In addition to adipocytes, macrophages also are known to accumulate excessive LDs in obesity, due to an imbalance between lipid synthesis and catabolism.^9^, ^10^ Lipid catabolism is mediated through both the canonical lipolytic pathway and lipophagy.^2^, ^11^

The classical lipolysis is a complex process that generates free fatty acids (FFA) from LD storage.^4^ Under basal conditions, adipose triglyceride lipase (ATGL) associates with LDs and hydrolyzes triacylglycerol (TAG) to diacylglycerol (DAG) and FFA, the so called basal lipolysis.^12^, ^13^, ^14^, ^15^ Activated by hormones, cAMP‐dependent protein kinase A (PKA) phosphorylates hormone‐sensitive lipase (HSL), which degrades DAG to monoacylglycerol (MAG) and FFA in a process known as stimulated lipolysis.^2^, ^14^ Monoacylglycerol lipase (MAGL) then converts MAG to glycerol and FFA.^16^ Both basal and stimulated lipolysis are highly regulated by LD‐associated proteins such as the perilipin (PLIN) family proteins.^17^ Among them, PLIN1 is an essential LD coating protein that prevents the basal lipolysis by restricting the access of ATGL to LDs.^17^, ^18^ The phosphorylated PLIN1, on the contrary, is required for the stimulated lipolysis, that phosphorylation of HSL is critically dependent on p‐PLIN1 for activity.^19^ Studies have shown that high fat diet (HFD)‐induced obesity is linked with DAG accumulation in cells such as adipocytes and hepatocytes.^4^, ^20^, ^21^ However, whether the dysregulated lipolysis with associated DAG build‐up plays a mechanistic role in the development of chronic inflammation in obesity is not fully understood. To determine if DAG accumulation drives the sustained inflammation and its downstream pathological effects, we tested Atglistatin (Atg), an ATGL inhibitor, to deplete the accumulated DAG and suppress its downstream inflammatory responses.

Autophagy is a cellular homeostasis process where cytosolic components such as proteins and damaged organelles are sequestered in double membrane‐bound autophagosomes and fused with lysosome for degradation.^22^ Recently, a macro‐autophagy (hereafter referred to as autophagy) has been demonstrated as a novel mechanism in lipid metabolism, named lipophagy.^23^ Lipophagy regulates LDs turnover and cells with defect in this process accumulate LDs.^24^, ^25^ Impaired autophagy in macrophages was associated with chronic inflammation in HFD‐induced obesity,^26^, ^27^ suggesting a correlation between LD accumulation and chronic inflammation. Further studies have shown that lipophagy functions in synergy with classical lipolysis in LD catabolism where lipolysis breaks down large LDs into small ones that are manageable for degradation by lipophagy.^11^ This homeostatic LD metabolism process is perturbed in obesity, leading potentially to a cycle of dysregulated lipolysis, impaired lipophagy and exacerbated LD accumulation, which may result in sustained chronic inflammation. To test this hypothesis, it is critical to probe the specific signalling pathways involved in this cycle of LD accumulation and inflammation.

Caspase recruitment domain‐containing protein 9 (CARD9) is an adaptor protein predominantly expressed in myeloid cells with an essential role in regulating innate and adaptive immune response.^28^, ^29^, ^30^ Activation of CARD9 signalling complex by PKCδ leads to up‐regulation of transcriptional factors p38 MAPK and NF‐κB and production of pro‐inflammatory cytokines such as IL‐6, TNFα and IL‐1β.^8^, ^30^, ^31^ Previously, we have shown that CARD9 was up‐regulated in peritoneal macrophages (PMs) of HFD‐fed obese mice.^8^ Moreover, fasting blood glucose level and insulin concentration were markedly elevated accompanied by impaired glucose disposal and insulin sensitivity in HFD‐fed WT mice.^8^ CARD9 KO significantly improved insulin sensitivity and glucose tolerance, suppressed plasma‐ and PM‐derived cytokines and protected against myocardial dysfunction. In the current study, we hypothesize that HFD feeding induces dysregulated lipolysis in PMs that in turn activates DAG‐PKCδ and CARD9‐dependent inflammation. This further impairs lipophagy leading to a positive signalling cycle of LD accumulation and sustained chronic inflammation. We will test that CARD9 KO interrupts this positive feedback loop and suppresses inflammation associated with HFD‐induced obesity.

## 2 MATERIALS AND METHODS

### 2.1 Experimental animals and HFD feeding regimen

C57BL/6 wild‐type (WT) and CARD9^−/−^ mice were fed with either normal rodent chow having 12% kcal as fat (normal diet, ND) or a HFD having 45% kcal as fat (D12451, Research Diets, NJ), starting from 4 to 6 weeks of age. Body weight was monitored over the 5‐months feeding period. At the end of 5‐months feeding, PMs were isolated for further studies. Mice were bred, weaned and housed in the animal care facility of University of Wyoming, College of Health Sciences. The procedures and methods were approved by Institutional Animal Care and Use Committee (IACUC) at the University of Wyoming and the investigation complied with the federal guidelines for the appropriate care of laboratory animals, Federal Law (89‐544, 91‐579) and all NIH regulations.

### 2.2 Atglistatin treatment

Atglistatin (Atg, Sigma‐Aldrich) was suspended in 0.5% DMSO in PBS as described previously.^32^ After 5‐months ND and HFD feeding, mice were randomly assigned into: (1) Atg treatment group with i.p. injection at a dose of 100 μmol/kg body weight; (2) vehicle (Veh) control group with i.p. injection of 0.5% DMSO in PBS using the same volume; (3) control group without any treatment. Both Atg and veh treatment were performed 8 h before PMs harvesting.^32^

### 2.3 Macrophage isolation and quantification

Peritoneal macrophages were harvested according to the standard protocols described previously.^8^, ^33^, ^34^ Briefly, 2 ml of 4% (wt/vol) thioglycollate (TG) broth (Sigma‐Aldrich), an eliciting agent, was injected i.p. to each mouse at the end of 5‐months HFD feeding. After 4 days of injection and 24 h of fasting, mice were sacrificed and peritoneal lavage performed using 10 ml ice‐cold DMEM/F‐12 media with 10% (vol/vol) heat‐inactivated foetal bovine serum and 1% (vol/vol) penicillin–streptomycin (Life Technologies). Ammonium‐chloride‐potassium lysing buffer (ACK) (Quality Biological) was used for lysing any red blood cell residuals present in the samples. To quantitate the number of macrophages in the peritoneal fluids, the total leukocyte count from the hemacytometer was multiplied by the percent of macrophages determined from differential cell count.^33^

### 2.4 Oil Red O (ORO) staining of LD

Once harvested, a total of 1–2 × 10^6^ cells in a complete DMEM/F12 medium were added to 35‐mm glass bottom cell culture dishes to form a monolayer.^33^ Then, the cells were allowed to adhere to the bottom of the dish by culturing for 2–3 h at 37°C under 5% CO_2_, with the non‐adherent cells removed by gently washing with warm PBS. After discarding the media, cells were fixed with paraformaldehyde (4%) for 20 min and subsequently washed and incubated with 60% (vol/vol) isopropanol for 15 s. Staining of PMs was followed with freshly prepared and filtered working solution of 60% Oil Red O (Sigma‐Aldrich) stock solution in isopropanol for 1 min at 37°C in dark. Cells were then de‐stained with 60% (vol/vol) isopropanol for 15 s and counterstained with DAPI (#62248, Invitrogen). Finally, Z‐stack images were taken using Zeiss LSM 710 confocal microscope with a magnification of 100×. Number of cells, number and size of LDs were calculated using ImageJ software (NIH) following established protocols (with no less than 100 cells examined from each group).^35^

### 2.5 Western immunoblotting analysis

The isolated macrophages were lysed in ice‐cold RIPA buffer. The supernatant was collected and stored at −80°C. Proteins were separated on 10%–15% sodium dodecyl sulphate‐polyacrylamide gel by electrophoresis and then transferred to nitrocellulose membrane (Pall BioTrace NT). After blocking with 3% bovine serum albumin containing 0.1% Tween 20 for 1 h at room temperature, target proteins were detected by incubating with primary antibodies at 4°C overnight. The following primary antibodies were used: from Cell Signalling Technology (CST), p‐p65 (1:500; #3033), p65 (1:1000; #8242), LC3BI/II (1:500; #2775), SQSTM1/p62 (1:1000; #5114), Becline‐1 (1:1000; #3738), p38 MAPK (1:1000; #9212), p‐p38 MAPK (1:2000; #4631), ATGL (1:1000; #2439), HSL (1:1000; #4107), p‐HSL (1:500; #4139), p‐PKCδ (1:500; #2055), PKCδ (1:1000; #2058), and GAPDH (1:5000; #5174); from Vala Sciences, PLIN1 (1:5000; #4854) and p‐PLIN1 (1:5000; #4856); and from Santa Cruz, P38IP (1:100; SC‐374665). After overnight incubation, membranes were probed with horseradish peroxidase‐conjugated secondary antibody (CST, #7074) for 1 h at room temperature. Signals were then detected with chemiluminescence reagent (LumiGlo; CST, #7003). Images were taken using ChemiDoc XRS imager (Bio‐Rad). Band intensity was determined by densitometry analysis using Quantity One® software and normalised using GAPDH as the loading control.

### 2.6 Measurement of diacylglycerol (DAG) and triacylglycerol (TAG) content

Concentrations of DAG and TAG were measured using DAG ELISA assay kit (#CEC038Ge, Cloud‐Clone Corp.) and picoProbe TAG assay kit (#ab178780, Abcam) according to manufacturer's instruction. Briefly, isolated macrophages were re‐suspended in fresh lysis buffer (# IS007, Cloud‐Clone Corp.) for DAG extraction or in triglyceride assay buffer for TAG extraction followed by ultra‐sonication until the solution was clear. DAG and TAG concentrations were determined from the supernatant of each sample after generating a standard curve using a microplate reader (SpectraMax 190, Molecular Devices). Results were normalised to the total protein concentration.

### 2.7 Immunofluorescence staining

The isolated PMs were cultured to obtain monolayer of cells as described previously.^33^, ^35^ This was followed by fixation with 4% paraformaldehyde for 20 min and blocking with a buffer containing PBS, 5% normal goat serum (#5425, CST), and 0.3% Triton™ X‐100 (#T8787, Sigma‐Aldrich) for 1 h. Then, the cells were incubated with primary antibody, LC3B (1:200; #3868, CST) diluted in PBS containing 1% BSA and 0.3% Triton X‐100 for overnight at 4°C. After washing three times in PBS, the samples were incubated with Alexa Flour 555 secondary antibody (#4413, CST) and BODIPY 493/503 (#D3922, Invitrogen) for 1 h followed by counterstaining with DAPI for 10 min. BODIPY 493/503 was used to stain LDs in green. Finally, Z‐stack images were taken using Zeiss LSM 980 confocal microscope. Images were analysed for co‐localization using ImageJ software (NIH) with at least 80 cells per group.

### 2.8 Statistical analysis

Statistical analysis was performed using GraphPad Prism Software (Version 8.0) by One‐Way anova followed by a Tukey test for post hoc analysis where appropriate. When comparing the means of two groups, unpaired t‐test was used. All measurements were repeated three times with at least three mice per group, and normality of data distribution was checked by Shapiro–Wilk test. For co‐localization study, Pearson's correlation coefficient was applied. Data were presented as mean ± SEM. Significant difference was determined at *p* < 0.05.

## 3 RESULTS

### 3.1 HFD feeding increased the numbers of LDs and PMs with LD in a CARD9‐dependent manner

**FIGURE 1:**
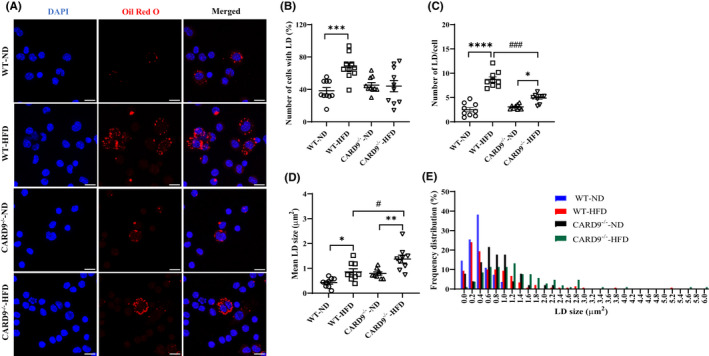
Lipid droplets (LDs) characterization in PMs after 5‐month HFD feeding. (A) Representative confocal images of ORO‐ (red, LDs) and DAPI‐(blue, nuclei) stained cells, scale bar: 10 μm. Quantitative analysis of the number of cells with LD (B), number of LD per cell (C), mean size of LD (μm^2^) (D) and frequency distribution of the size of LD (E). Results were representative of at least three independent experiments. Data were presented as mean ± SEM, *n* = 9‐10/group; *p < 0.05, **p < 0.01, ***p < 0.001, ****p < 0.0001, HFD versus ND; ^#^ *p* < 0.05, ^###^ *p* < 0.001, CARD9^−/−^ versus WT.

As shown in Figure S1A, HFD feeding resulted in a continuous increase in body weight in both WT and CARD9^−/−^ mice with significant difference after 1 month compared with their respective controls. The percentage yield of PMs in the HFD‐fed WT and HFD‐fed CARD9^−/−^ mice were increased by 95% (*p* < 0.0001) and 93% (*p* < 0.0001) compared with their respective control groups without TG treatment (Figure S1B). To visualize the accumulation of LD following 5‐month HFD feeding, PMs were stained with Oil red O (ORO). Representative confocal microscopy images of ORO‐ and DAPI‐stained cells are shown in Figure 1A. Quantitative analysis of the percentage of cells with LD revealed a significant increase in the HFD‐fed WT mice by 67.6% versus 38.3% in the ND‐fed WT mice (*p* < 0.001). CARD9 KO ameliorated this increase with no significant difference between the HFD‐ and ND‐fed CARD9^−/−^ mice (Figure 1B). The number of LDs per cell also showed a marked increase in the HFD‐fed WT mice and a slight but significant increase in the HFD‐fed CARD9^−/−^ mice compared with their respective ND‐fed groups (Figure 1C). Compared with the HFD‐fed WT mice, CARD9 KO significantly reduced the number of LDs per cell by about 44.8% (*p* < 0.001) after HFD feeding. Interestingly, the mean LD size showed a marked increase from 0.42 μm^2^ in the ND‐fed WT mice to 0.86 μm^2^ in the HFD‐fed WT mice (*p* < 0.05) and 0.79 μm^2^ in the ND‐fed CARD9^−/−^ mice to 1.34 μm^2^ in the HFD‐fed CARD9^−/−^ mice (*p* < 0.01) (Figure 1D). There is also a significant increase in LD size in the HFD‐fed CARD9^−/−^ mice compared with that of the HFD‐fed WT mice. Size distribution of LDs revealed that HFD feeding shifted the size of LDs to the right in both WT and CARD9^−/−^ mice (Figure 1E). These results indicated that CARD9 KO reduced the number of LDs and the number of cells with LDs and increased the mean size of LDs.

### 3.2 HFD feeding dysregulated LD lipolysis in PMs in a CARD9‐dependent manner

**FIGURE 2:**
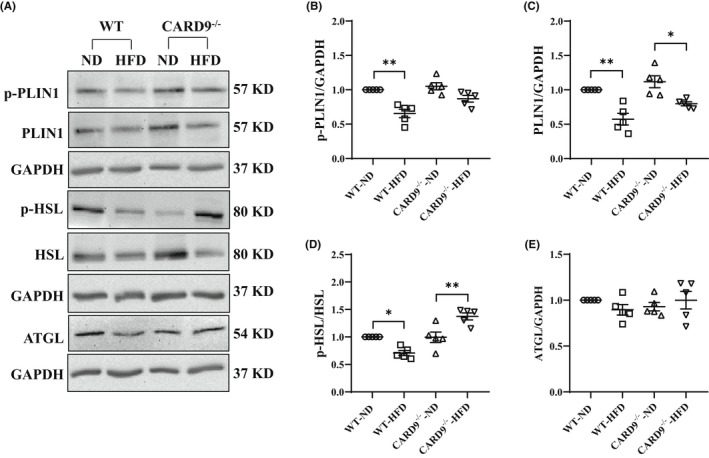
Lipolytic protein expression in PMs after 5‐month HFD feeding. (A) Representative Western blots of PLIN1, p‐PLIN1, HSL, p‐HSL, and ATGL. Densitometric analysis of p‐PLIN1 (B), PLIN1 (C), p‐HSL (D), and ATGL (E). GAPDH was used as the loading control. Results were representative of at least three independent experiments. Data were presented as mean ± SEM, *n* = 5/group; *p < 0.05, **p < 0.01, HFD versus ND.

As lipolysis is essential to the dynamics of LD accumulation and metabolism, lipolytic proteins were assessed. Western immunoblotting analysis was performed from lysis of PMs on protein expression of phosphorylated‐perilipin‐1 (p‐PLIN1), PLIN1, phosphorylated‐hormone‐sensitive lipase (p‐HSL), HSL and adipose triglyceride lipase (ATGL). Representative blots are shown in Figure 2A. Quantitative analysis revealed that the relative abundance of p‐PLIN1 and p‐HSL, which mediate stimulated lipolysis, were markedly reduced in the HFD‐fed WT mice compared with the ND‐fed WT mice (Figure 2B,D). CARD9 KO ameliorated the reduction of p‐PLIN1 (Figure 2B), but significantly increased p‐HSL (Figure 2D). HFD feeding also markedly reduced PLIN1 in both HFD‐fed WT and CARD9^−/−^ mice compared with their respective ND‐fed controls (Figure 2C). There was no significant difference in ATGL level among all the experimental groups (Figure 2E). Taken together, these results suggest that the basal lipolysis (depending on PLIN1) is enhanced in both HFD‐fed WT and CARD9^−/−^ mice but the stimulated lipolysis (depending on p‐PLIN1) is only down regulated in the HFD‐fed WT mice. CARD9 KO restored the stimulated lipolysis.

### 3.3 HFD feeding increased DAG, but CARD9 KO and Atglistatin treatment ameliorated the increase of DAG

**FIGURE 3:**
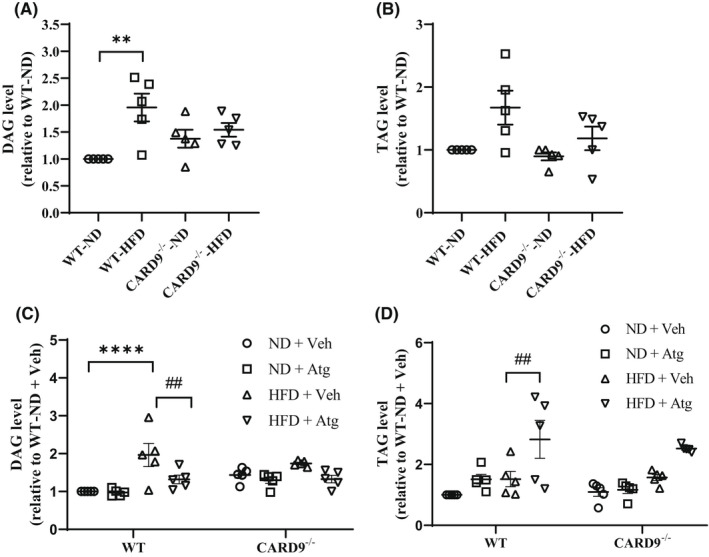
Percentage DAG and TAG content in PMs after 5‐month HFD feeding and Atg treatment. Quantitative analysis of DAG (A), TAG (B), DAG with Atg or Veh (C), and TAG with Atg or Veh (D). Results were representative of at least three independent experiments. Data were presented as mean ± SEM, *n* = 5/group; **p < 0.01, ****p < .0001, HFD vs. ND; ^##^ *p* < 0.01, Atg versus Veh.

Next, we examined if the reduction in PLIN1 (suggesting an increase from TAG to DAG), p‐PLIN1 and p‐HSL (suggesting a decrease from DAG to MAG) observed in the HFD‐fed WT mice leads to DAG accumulation. As shown in Figure 3A, HFD feeding significantly increased DAG by about 2‐fold compared with the ND‐fed WT mice (*p* < 0.01). CARD9 KO, however, normalized the level of DAG compared with the ND‐fed CARD9^−/−^ mice. There is no significant difference in TAG among all the groups (Figure 3B). To further determine if the dysregulated basal lipolysis is the cause of the increase in DAG in the HFD‐fed WT mice, we treated the mice with Atg, an inhibitor of ATGL. This would inhibit the conversion of TAG to DAG and normalize the level of DAG. As shown in Figure 3C, Atg treatment significantly reduced DAG by 33.2% compared with the veh‐treated WT mice (*p* < 0.01). The administration of either veh or Atg did not cause any significant change to the level of DAG in the HFD‐fed CARD9^−/−^ mice (Figure 3C). The level of TAG was markedly increased following Atg treatment by about 1.9‐fold compared with the veh‐treated WT mice (Figure 3D) further confirming the inhibition of TAG to DAG. There was no significant change in TAG among all the CARD9 KO groups. These results indicated that the dysregulated lipolysis, both conversion of TAG to DAG and DAG to MAG, in the HFD‐fed WT mice resulted in DAG accumulation, and mechanistically CARD9 deficiency or Atg treatment normalized the level of DAG.

### 3.4 HFD feeding induced but CARD9 KO and Atg treatment ameliorated the activation of PKCδ, NF‐kB and p38 MAPK

**FIGURE 4:**
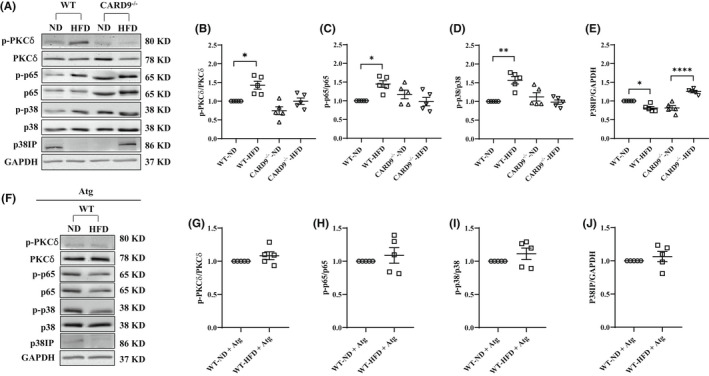
Protein expression of PKCδ, NF‐κB and p38 MAPK in PMs after 5‐month HFD feeding and Atg treatment. (A and F) Representative Western blots. Densitometric analysis of p‐PKCδ/PKCδ (B), p‐p65/p65 (C), p‐p38/p38 (D), p38IP/GAPDH (E) and with Atg (G–J). GAPDH was used as the loading control. Results were representative of at least three independent experiments. Data were presented as mean ± SEM, *n* = 5/group; *p < 0.05, **p < 0.01, ****p < 0.0001, HFD versus ND.

Next, we tested if the dysregulated lipolysis and the increased DAG accumulation in the HFD‐fed WT mice activates PKCδ which further activates CARD9‐dependent NF‐κB and p38 MAPK inflammatory pathways as suggested.^28^, ^30^, ^36^ Western immunoblotting analysis was performed on protein expression of PKCδ, p‐PKCδ, p38 MAPK, p‐p38 MAPK, p‐p65 and p65 with representative blots shown in Figure 4A,F. HFD feeding markedly increased the ratio of p‐PKCδ/ PKCδ by 1.4‐fold in the WT mice compared with the ND‐fed control group (Figure 4B, *p* < 0.05), with no significant difference among the CARD9^−/−^ groups. HFD feeding also significantly increased the ratio of p‐p65/p65 (Figure 4C) and p‐p38/p38 (Figure 4D) by 1.5‐ (*p* < 0.05) and 1.6‐fold (*p* < 0.01) respectively compared with their ND‐fed groups. CARD9 KO ameliorated these changes. Further, Atg treatment suppressed the increase in p‐PKCδ/ PKCδ, p‐p65/ p65 and p‐p38/p38 MAPK in the HFD‐fed WT mice (Figure 4G–I). These data indicated that the dysregulated lipolysis in the HFD‐fed WT mice activated the DAG‐PKCδ pathway which further activated the CARD9 inflammatory signalling. CARD9 KO and Atg treatment ameliorated these inflammatory responses.

### 3.5 HFD feeding reduced but CARD9 KO and Atg treatment restored p38IP

It was reported that p‐p38 MAPK interacts with the p38‐interacting protein (p38IP) resulting in suppression of p38IP‐initiated autophagosome formation, an alternative autophagy pathway.^37^ To test if the activated CARD9 signalling inhibits autophagy/lipophagy, Western immunoblotting analysis was performed on protein expression of p38IP. As shown in Figure 4E, p38IP was significantly down regulated by 0.8‐fold (*p* < 0.05) in the HFD‐fed WT mice compared with the ND‐fed WT mice. CARD9 KO markedly increased the level of p38IP by 1.6‐fold (*p* < 0.0001) while Atg treatment restored p38IP expression (Figure 4J), suggesting protected autophagosome formation and potentially lipophagy.

### 3.6 HFD feeding impaired, CARD9 KO and Atg treatment restored autophagy signalling

**FIGURE 5:**
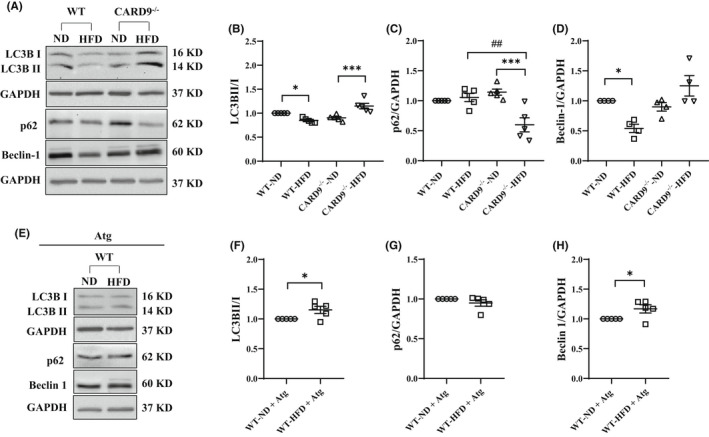
Autophagy signalling in PMs after 5‐month HFD feeding and Atg treatment. (A and E) Representative Western blots. Densitometric analysis of LC3BII/I (B), p62/GAPDH (C), Beclin‐1/GAPDH (D) and with Atg (F–H). GAPDH was used as the loading control. Results were representative of at least three independent experiments. Data were presented as mean ± SEM, *n* = 5/group; *p < 0.05, ***p < 0.001, HFD versus ND; ^##^ *p* < 0.01, CARD9^−/−^ versus WT.

As defects in macrophage autophagy were associated with chronic inflammation in obesity,^26^, ^27^ next, we tested if CARD9‐dependent inflammation alters autophagy in PMs of HFD‐fed WT mice. Western immunoblotting analysis was performed on protein expression of LC3BII/I, p62 and Beclin‐1, markers of autophagy initiation and maturation with representative blots shown in Figure 5A,E. HFD feeding significantly reduced LC3BII/I ratio by 26% (Figure 5B, *p* < 0.05) and Beclin‐1 by 46% (Figure 5D, *p* < 0.05) in the HFD‐fed WT mice compared with the ND‐fed WT mice with no significant change to p62 (Figure 5C). CARD9 KO restored LC3BII/I (Figure 5B), Beclin‐1 (Figure 5D) and reduced p62 (Figure 5C). Interestingly, Atg treatment markedly increased LC3BII/I and Beclin‐1 in the HFD‐fed WT mice compared with the ND‐fed WT mice without significant change to p62 (Figure 5F–H). These results indicated that the activated DAG‐PKCδ and CARD9 signalling resulted in altered autophagy signalling in the HFD‐fed WT mice, and CARD9 or Atg treatment restored autophagy.

### 3.7 HFD feeding impaired but CARD9 KO and Atg treatment restored lipophagy‐mediated degradation of LDs

**FIGURE 6:**
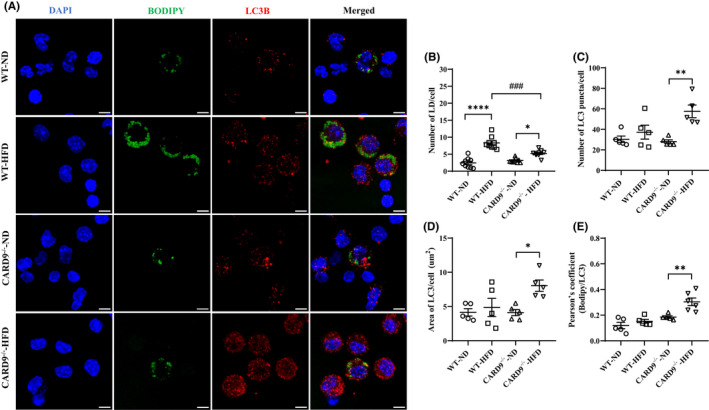
Immunofluorescence staining of LC3B and LDs in PMs after 5‐month HFD feeding. (A) Representative images of co‐immunostaining for LC3B and LDs, scale bar: 5 μm. (B) The number of LDs per cell, (C) The number of LC3B puncta per cell, (D) Area of LC3B puncta per cell (μm^2^), (E) Pearson's correlation coefficient corresponding to the fraction of green pixels (LDs) that overlap with red pixels (LC3B). Results were representative of at least three independent experiments. Data were presented as mean ± SEM, *n* = 5–9/group; **p < 0.01, ****p < 0.0001, HFD versus ND; ^###^ *p* < 0.001, CARD9^−/−^ versus WT.

Next, we tested if the autophagic protein LC3B overlaps with LDs to serve as a lipophagic signalling. PMs were harvested for immunofluorescence staining where anti‐LC3B antibody and BODIPY were used to stain autophagosome and LDs, respectively. Representative confocal microscopy images are presented in Figure 6A. As shown in Figure 6B,C, HFD feeding markedly increased the number of LD per cell without significant change to the number of LC3B^+^ autophagy puncta per cell in the HFD‐fed WT mice compared with the ND‐fed WT mice. The HFD‐fed CARD9^−/−^ mice also exhibited a slight but significant increase in the number of LD per cell compared with the respective ND‐fed mice, similar to ORO staining results (Figure 6B vs. Figure 1C). The number of LC3B^+^ puncta per cell was markedly increased by about 2‐fold (*p* < 0.01) in the HFD‐fed CARD9^−/−^ mice compared with the respective ND‐fed mice (Figure 6C). The total area of LC3B^+^ staining per cell, corresponding to the total LC3B expression, was also markedly increased by about 2‐fold in the HFD‐fed CARD9^−/−^ mice compared with the respective ND‐fed group (Figure 6D, *p* < 0.05), with no significant difference among the WT groups. The incidence of LC3B co‐localization with LDs was evaluated by Pearson's correlation analysis. As shown in Figure 6E, the HFD‐fed CARD9^−/−^ mice exhibited a 1.7‐fold increase in the correlation coefficient compared with the respective ND‐fed group (*p* < 0.01), with no significant difference among the WT groups.

**FIGURE 7:**
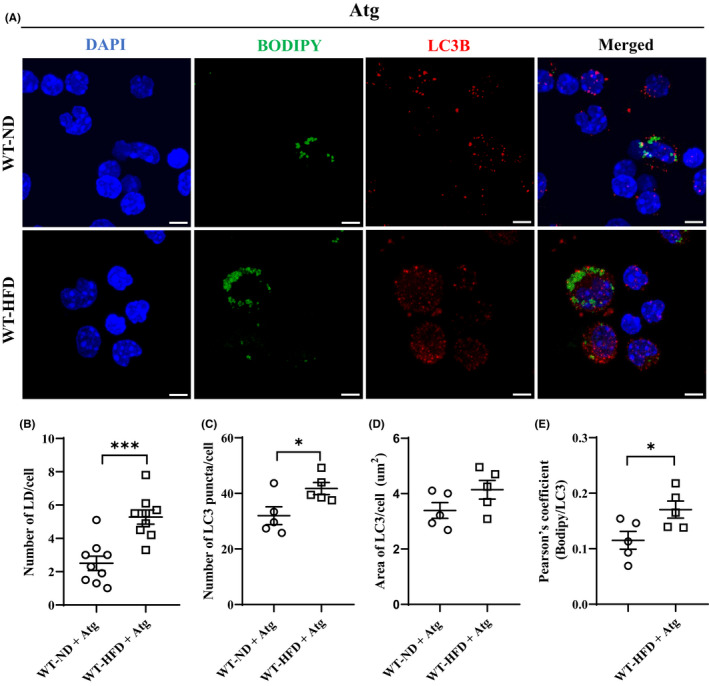
Immunofluorescence staining of LC3B and LDs in PMs after 5‐month HFD feeding with Atg treatment. (A) Representative images of co‐immunostaining for LC3B and LDs, scale bar: 5 μm. (B) The number of LDs per cell, (C) The number of LC3B puncta per cell, (D) Area of LC3B puncta per cell (μm^2^), (E) Pearson's correlation coefficient corresponding to the fraction of green pixels (LDs) that overlap with red pixels (LC3B). Results were representative of at least three independent experiments. Data were presented as mean ± SEM, *n* = 5–9/group; *p < 0.05, ***p < 0.001, HFD versus ND.

To assess if Atg treatment enhances lipophagy, WT mice were treated with Atglistatin as in the Material and Methods section. Representative confocal microscopy images are presented in Figure 7A. As shown in Figure 7B, Atg treatment did not affect the number of LD per cell. However, Atg treatment significantly increased the number of LC3B puncta per cell (Figure 7C) and the Pearson's coefficient (Figure 7E), with no change to the area of LC3B per cell (Figure 7D). The number of LDs per cell was still significantly higher in the treated HFD‐fed WT mice compared with the ND‐fed WT mice, but was significantly reduced from that of the untreated HFD‐fed WT mice as in Figure 6B. Altogether, these data indicated that CARD9 KO and Atg treatment increased the number of LC3B^+^ autophagosome, enhanced lipophagy therefore lipophagy‐mediated LD degradation.

## 4 DISCUSSION

**SCHEME 1:**
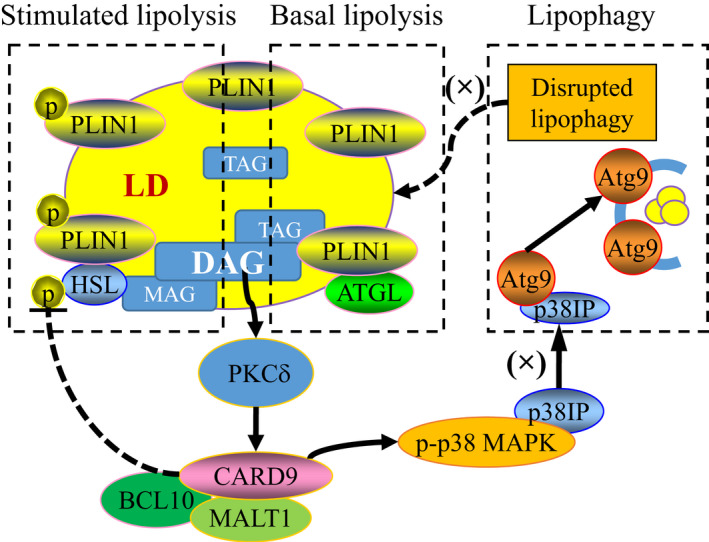
HFD‐induced down‐regulation of PLIN1, p‐PLIN1 and p‐HSL activates DAG/PKCδ signalling, leading to activation of CARD9 signalling complex. Activated CARD9 signalling phosphorylates p38 MAPK, disrupts p38IP‐dependent lipophagy and exacerbates lipid accumulation in macrophages. Solid arrow—direct mechanistic link; dashed arrow and line—speculated link; (×)—blocked pathway.

Obesity is hallmarked with increased lipid accumulation in various tissues.^38^, ^39^ It is associated with a constellation of metabolic disorders with chronic inflammation.^25^, ^40^ Our previous study have shown that CARD9 deficiency attenuated HFD‐induced insulin resistance, glucose intolerance and PM‐derived inflammatory cytokines.^8^ Here, we report that HFD feeding dysregulated lipolysis and activated DAG‐PKCδ signalling which further activated CARD9‐dependent inflammation and impaired lipophagy, leading to LD accumulation. The excessive LD accumulation and impaired LD metabolism may sustain a chronic inflammation underlying HFD‐induced obesity. CARD9 and Atg treatment interrupted this positive feedback cycle and provided protection against HFD‐induced chronic inflammation and associated metabolic abnormalities as depicted in Scheme 1.

There are several key findings in the current study. First, CARD9 KO reduced the number of PMs harbouring LDs and the number of LDs in PMs following HFD‐feeding. Interestingly, these LD‐containing PM cells from the HFD‐fed CARD9^−/−^ mice showed larger LD with mean droplet size up to 6 μm^2^. A previous report suggested that the size of LD is an essential physical characteristic that governs the process of LD metabolism following diet‐induced obesity in hepatocytes.^11^ The authors were able to show that while the lipolytic enzymes targeted large LDs, the lipophagic machinery could only encompass the small ones preferentially for degradation. Another study also suggested that the size of a cargo destined for catabolism through autophagy may be a crucial factor governing degradation by the autophagic machinery.^41^ The size‐dependent LD catabolism might explain our observation of larger LDs in the HFD‐fed CARD9^−/−^ mice despite improved lipolysis and enhanced lipophagy, as also suggested previously.^42^, ^43^

Secondly, CARD9 KO and Atg treatment restored the dysregulated lipolysis following HFD‐feeding. Previously, it was demonstrated that two‐months HFD feeding increased basal lipolysis and reduced stimulated lipolysis in adipocytes.^4^ The lipolytc proteins p‐HSL and PLIN1 were found to be compromised, leading to DAG build‐up.^44^ Consistent with these studies, we observed a dysregulated lipolysis in PMs of the HFD‐fed WT mice with marked reduction of p‐PLIN1 and p‐HSL. Among the PLIN family of LD coating proteins, PLIN1 is implicated in the TAG hydrolysis as a ‘gate‐keeper’ during basal lipolysis by negatively regulating TAG to DAG conversion via ATGL.^17^ Our study revealed that PLIN1 is significantly suppressed in PMs of both WT and CARD9^−/−^ mice after HFD feeding, suggesting enhanced conversion from TAG to DAG. This is in agreement with previous reports in other cell types such as adipocytes and hepatocytes.^4^, ^20^ CARD9 KO and Atg treatment improved stimulated lipolysis therefore prevented DAG accumulation.

Thirdly, CARD9 KO and Atg treatment suppressed the activation of DAG‐PKCδ and its downstream inflammatory signalling. Previous studies with HFD‐induced obesity and HSL^−/−^ mice have demonstrated DAG accumulation in cells and tissues.^20^, ^21^ Yet the mechanistic connection of DAG build‐up to any consequential inflammatory response has not well documented. As DAG mediates various intracellular signalling pathways through the activation of DAG‐sensing proteins,^45^ we assessed if PKCδ is activated in PMs of HFD‐fed WT mice, as phosphorylation of PKCδ is essential for the activation of CARD9, and NF‐kB and p38 MAPK.^30^, ^36^, ^46^ Our results that CARD9 KO and Atg treatment ameliorated HFD feeding‐induced increase in p‐PKGδ/PKGδ suggested that CARD9 activation may propel a sustained chronic inflammation possibly due to DAG‐PKCδ activation.

Lastly, our study suggested an underlying mechanism where the dysregulated lipolysis with CARD9 activation impaired lipophagy which was restored in CARD9^−/−^ mice or by treatment with Atg. Obesity is characterised by defective autophagy/lipophagy in a cell type‐dependent manner: adipocytes and pancreatic β cells exhibited increased autophagy,^47^, ^48^ but hepatocytes showed the opposite regulation.^23^ It was reported that HFD‐induced obesity is linked with reduced autophagy in PMs resulting in pro‐inflammatory polarization.^27^ Our data on the co‐localization of LC3B^+^ autophagosome with LD in CARD9 KO or Atg treatment mice suggested a potential mechanistic link between obesity‐induced inflammation and defective autophagy. Previous studies have shown that the phosphorylated p38 MAPK interacts with p38IP, preventing its association with Atg9 and negatively impairing autophagy. Based on our observation on the decrease of p‐p38/p38 and increase of p38IP in the HFD‐fed CARD9^−/−^ mice, we speculate that p38IP, a potential partner to Atg9 trafficking and autophagosome formation, as an alternative autophagy pathway, may have mediated the CARD9‐dependent impairment of lipophagy.^37^ Future mechanistic studies are warranted to fully understand how CARD9 mediates the interaction between p38IP and Atg9.

In conclusion, our study demonstrated that HFD‐induced obesity is associated with dysregulated LD lipolysis in PMs, resulting in activation of DAG‐PKCδ signalling and CARD9‐dependent inflammation and impairment of lipophagy. In turn, the impaired lipophagy may result in excessive lipid accumulation and further dysregulation of lipolysis, sustaining a potential positive feedback cycle underlying obesity‐associated chronic inflammation. CARD9 deficiency and Atg treatment alleviated the chronic inflammation by restoring the dysregulated lipolysis and enhancing the impaired lipophagy. Our findings suggest that CARD9 may server as a potential therapeutic target in suppressing chronic inflammation associated with obesity and related metabolic disorders characterized by defective lipid metabolism.

## AUTHOR CONTRIBUTIONS

**Yohannes Getiye:** Data curation (lead); formal analysis (lead); methodology (lead); visualization (lead); writing – original draft (lead). **Tatiana Angel Rice:** Data curation (supporting); formal analysis (equal); investigation (supporting). **Brandon D. Phillips:** Data curation (equal); formal analysis (supporting). **Daniel Fidel Carrillo:** Data curation (equal); formal analysis (equal); investigation (supporting). **Guanglong He:** Conceptualization (lead); funding acquisition (lead); project administration (lead); resources (lead); supervision (lead); writing – review and editing (lead).

## CONFLICT OF INTEREST

The authors declared that they have no conflict of interest.

## Supporting information


**FigureS1** Supporting Information

## Data Availability

The data sets supporting the findings of the current study are available from the corresponding author upon reasonable request.
