# Supplementary material for: Dysregulated lipolysis and lipophagy in lipid droplets of macrophages from high fat diet‐fed obese mice
Source: J Cell Mol Med. 2022 Aug 13;26(18):4825–36. doi: 10.1111/jcmm.17513 (PMC9465182; doi:10.1111/jcmm.17513)

Supplemental Figure

The Dysregulated Lipolysis and Lipophagy in Lipid droplets Of Macrophages From High Fat Diet-fed Obese Mice

Yohannes Getiye [a], Tatiana Rice [a], Brandon D. Phillips [a], Daniel Carrillo [a], Guanglong He [a,*]

Supplemental Figure 1

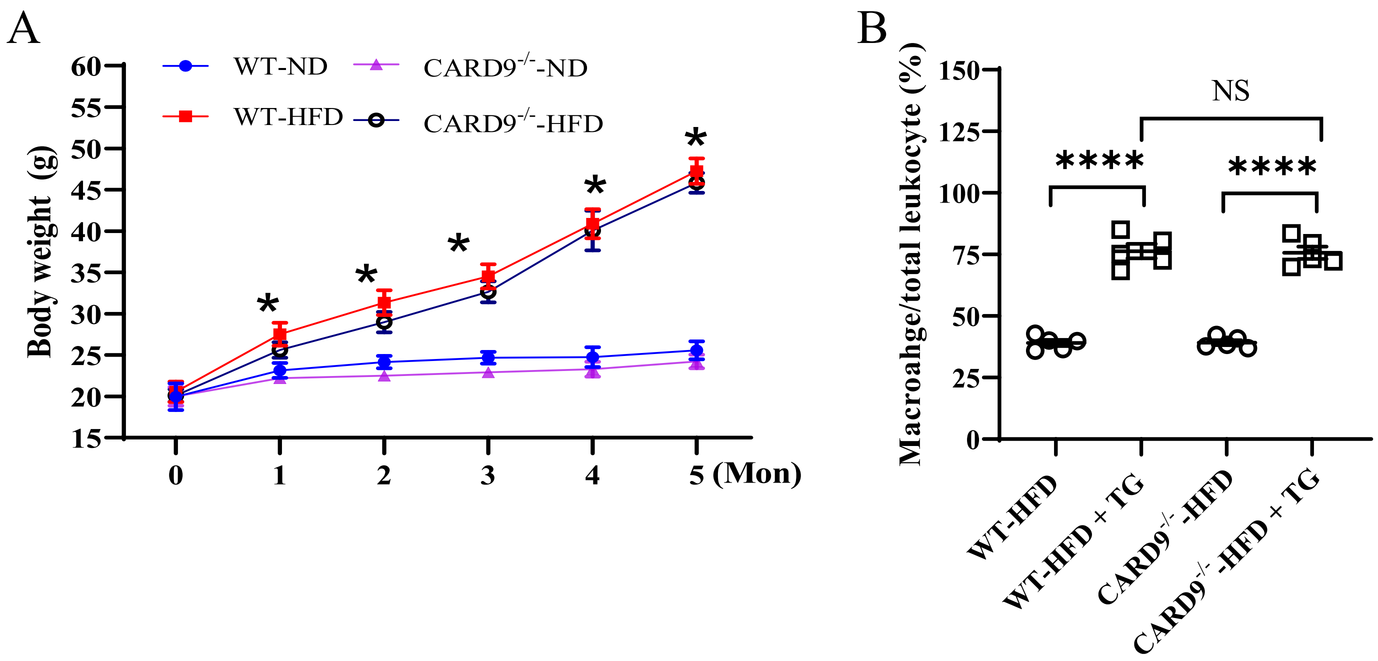

Supplement: Supplementary file 1 — FigureS1 Supporting Information [file JCMM-26-4825-s001.pdf]
